# Colonic Schistosomiasis Presenting with Findings of Inflammatory Bowel Disease

**DOI:** 10.4269/ajtmh.21-0312

**Published:** 2021-07-08

**Authors:** Yohannes Birhanu, Mesfin Asefa, Amir Sultan

**Affiliations:** ^1^ Department of Internal Medicine, Division of Gastroenterology, College of Health Sciences, Addis Ababa University, Addis Ababa, Ethiopia;; ^2^ Girum Hospital, Addis Ababa, Ethiopia;; ^3^ Department of Pathology, Saint Paul Hospital Millennium Medical College, Addis Ababa, Ethiopia

**Figure 1.**
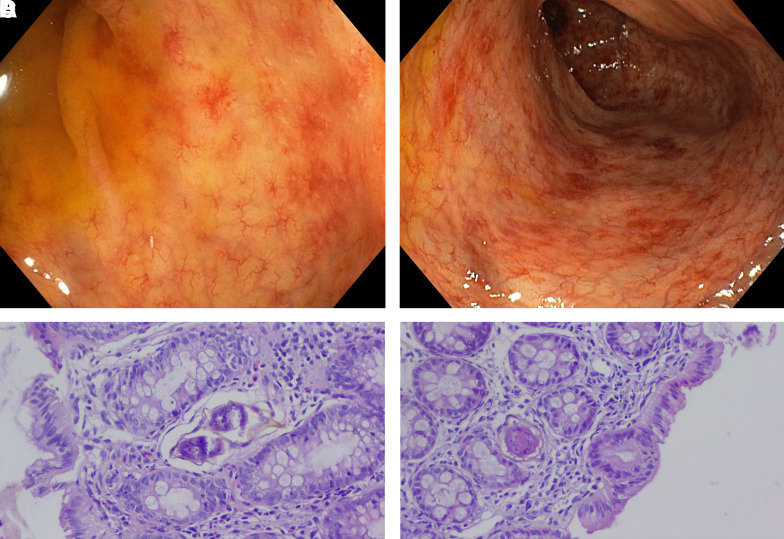
(**A**) Colonic mucosa with surface hyperemia and inflammation. (**B**) Colonic nodular lesions with surrounding hyperemia. (**C**) Egg in colonic tissue. (**D**) Granulomas in colonic mucosa. This figure appears in color at www.ajtmh.org.

A 19-year-old man presented to our hospital in Addis Ababa (Ethiopia) with an 8-month history of recurrent abdominal pain, loss of appetite, and occasional blood in the stool. Physical and basic laboratory examinations, including liver enzymes, were unremarkable. The stool examination was negative for ova and parasites. The abdominal ultrasonography results were normal. A colonoscopy was performed with a working diagnosis of inflammatory bowel disease. The examination revealed a patchy mucosal erythema with scattered nodularity and multiple vascular lesions in the ascending colon, sigmoid, and rectum (Figure 1A and B). Colon biopsies revealed multiple *Schistosoma* eggs with surrounding eosinophilic inflammation and granuloma (Figure 1C and D). It was noted that the patient resides in Bahir Dar, a lakeside city in northern Ethiopia endemic for schistosomiasis. On inquiry, the patient reported a history of swimming in the lake and washing clothes in the river.

The patient was treated with one dose of oral praziquantel 40 mg/kg. He reported complete resolution of symptoms during a clinic visit 2 weeks later. Informed consent was received from this patient to publish his case.

Schistosomiasis is a common helminth-related disease that frequently affects the liver or bladder, depending on the species. Intestinal schistosomiasis, usually caused by *S. mansoni*, occurs as eggs migrate through the intestinal wall, thus provoking mucosal granulomatous inflammation.^1^
